# Supplementary material for: Simulation of Figures of Merit for Barristor Based on Graphene/Insulator Junction
Source: Nanomaterials (Basel). 2022 Aug 31;12(17):3029. doi: 10.3390/nano12173029 (PMC9457586; doi:10.3390/nano12173029)
Supplement: Supplementary file 1 [file nanomaterials-12-03029-s001.zip › nanomaterials-1839401_supplementary.pdf]

Supplementary materials of "Simulation of Figures of Merit for Barristor based on graphene/insulator junction."

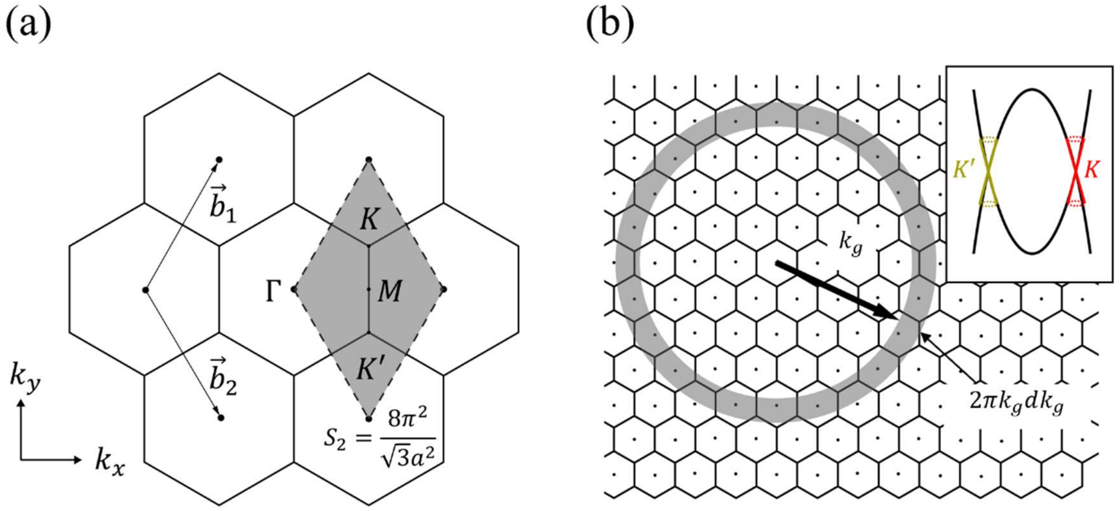

Figure S1. (a) Reciprocal lattice space of graphene crystal. (b) The calculation of 2D density of states of graphene crystal. Lattice points within grey areas are encountered within corresponding area. (inset) Linear dispersion of energy band diagram of graphene near K points.

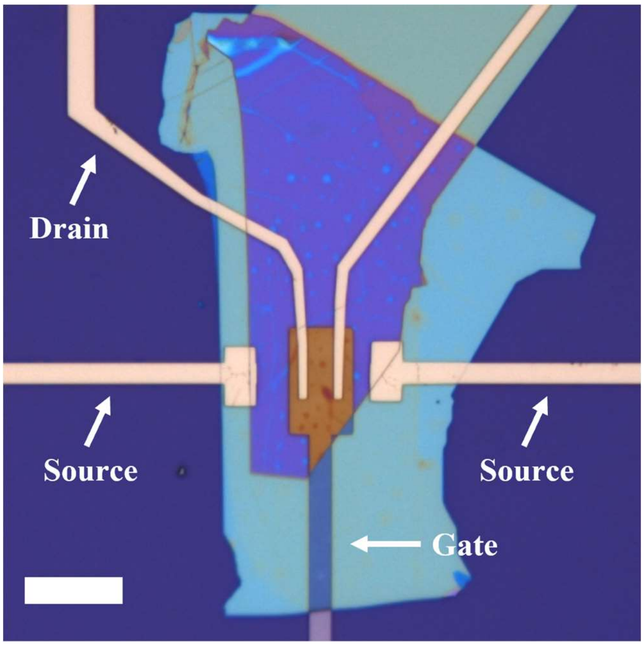

Figure S2. Optical microscope image of a GIM device measured in this experiment. Source, drain, and gate electrodes are marked with white arrows. Top and bottom hBN thickness is 64, 62nm each. Scale bar in the lower left corner is 20um.

Supplementary Software File. Simulation MATLAB code is provided as "S1 MATLAB code.zip"
